# Design of an Automated Flow Injection-Chemiluminescence Instrument Incorporating a Miniature Photomultiplier Tube for Monitoring Picomolar Concentrations of Iron in Seawater

**DOI:** 10.1155/JAMMC.2005.37

**Published:** 2005

**Authors:** Andrew R. Bowie, Eric P. Achterberg, Simon Ussher, Paul J. Worsfold

**Affiliations:** 1 Antarctic CRC University of Tasmania Private Bag 80 Tasmania Hobart 7001 Australia; 2 School of Earth Ocean and Environmental Sciences Plymouth Environmental Research Centre University of Plymouth Plymouth PL4 8AA UK

## Abstract

A flow-injection (FI)-based instrument under LabVIEW control for monitoring iron in marine waters is described. The instrument incorporates a miniature, low-power photomultiplier tube (PMT), and a number of microelectric and solenoid actuated valves and peristaltic pumps. The software allows full control of all flow injection components and processing of the data from the PMT. The optimised system is capable of 20 injections per hour, including preconcentration and wash steps. The detection limit (3 sd of the blank) is 21 pM at sea and the linear range is 21–2000 pM with a 60-second sample load time. Typical precision between replicate FI peaks is 5.9 ± 3.2 % (*n* =4) over the linear range.


Journal of Automated Methods & Management in Chemistry, **2005** (2005), no. 2, 37–43
© 2005 Hindawi Publishing Corporation

### Design of an Automated Flow Injection-Chemiluminescence Instrument Incorporating a Miniature Photomultiplier Tube for Monitoring Picomolar Concentrations of Iron in Seawater

Andrew R. Bowie,[1] Eric P. Achterberg,[2] Simon Ussher,[2] and Paul J. Worsfold[2]

[1] *Antarctic CRC, University of Tasmania, Private Bag 80, Hobart, Tasmania 7001, Australia*
[2] *School of Earth, Ocean and Environmental Sciences, Plymouth Environmental Research Centre, University of Plymouth, Plymouth PL4 8AA, UK*

*Received 23 September 2004; Accepted 23 September 2004*

A flow-injection (FI)-based instrument under LabVIEW control for monitoring iron in marine waters is described. The instrument incorporates a miniature, low-power photomultiplier tube (PMT), and a number of microelectric and solenoid actuated valves and peristaltic pumps. The software allows full control of all flow injection components and processing of the data from the PMT. The optimised system is capable of 20 injections per hour, including preconcentration and wash steps. The detection limit (3 sd of the blank) is 21 pM at sea and the linear range is 21–2000 pM with a 60-second sample load time. Typical precision between replicate FI peaks is 5.9 ± 3.2% ($n = 4$) over the linear range.

### 1. INTRODUCTION

Iron is an important parameter to determine in seawater because of its role in photosynthetic processes [1], ocean productivity [2], and hence global carbon cycling [3]. Open-ocean concentrations of dissolved iron(II+III) are in the range of 50–700 pM [4] and are typically depleted in surface waters and elevated at depth. Iron in seawater can be determined in the laboratory using isotope dilution HR-ICP-MS after coprecipitation [5] or HR-ICP-MS after solid-phase extraction [6], GFAAS after solvent extraction [7], flow injection (FI) with chemiluminescence (CL) [8, 9], spectrophotometric [10] detection or cathodic stripping voltammetry [11]. Current oceanographic studies however require the determination of iron at sea in real time and this necessitates the use of portable, shipboard instrumentation, for which FI techniques are ideally suited.

This paper describes the design and performance characteristics of a fully automated and portable FI instrument with CL detection for real-time monitoring of iron at sea. The system incorporates a low power (5 V) photomultiplier tube (PMT), an immobilised chelating resin for analyte preconcentration and luminol chemistry for detection. Iron(II) can be determined directly by its enhancing effect on the luminol reaction and "total" iron(II+III) can be determined after acidification and sample reduction steps. A graphical programming environment (LabVIEW, http://sine.ni.com/labview) facilitates the design of a virtual instrument with a fully flexible user interface for instrument control and data acquisition.

Correspondence and reprint requests to Paul J. Worsfold, School of Earth, Ocean and Environmental Sciences, Plymouth Environmental Research Centre, University of Plymouth, Plymouth PL4 8AA, UK; E-mail: pworsfold@plymouth.ac.uk.

### 2. EXPERIMENTAL

### 2.1. Reagents and standards

All chemicals were obtained from Merck BDH (Crown Scientific, Kingston, Australia), unless otherwise stated. Labware was cleaned by soaking in successive baths of hot 5% (v/v) micro-detergent (Decon) for 24 hours, 6 M HCl (AnalaR) for 1 week, and 2 M $HNO_3$ (AnalaR) for 1 week, with thorough rinses using doubly deionised water (DIW, 18.2 $M\Omega\,cm^{-1}$) between each step. Sample handling was carried out in a class-100 laminar flow hood. High purity quartz distilled (Q-) HCl, $HNO_3$, ammonia, and acetic acid were obtained from Seastar (Baseline grade, Sidney, BC, Canada).

Iron(II) standards were prepared daily in 0.1 M Q-HCl from $Fe(NH_4)_2(SO_4)_2{\cdot}6H_2O$. Luminol (Sigma, Perth, Australia) ($1 \times 10^{-5}$ M) was prepared in 0.1 M $Na_2CO_3$ by dilution of a 0.01 M stock, adjusted to pH 12.2 with 2 M NaOH, and passed through a Chelex-100 (Sigma) chelating resin column just prior to use. Ammonium acetate sample buffer (0.4 M) was prepared from a 2 M stock and adjusted to pH 5.5 with Q-acetic acid. An iron(III) reducing agent of 100 $\mu$M $Na_2SO_3$ (extra pure) was prepared from a 0.4 M

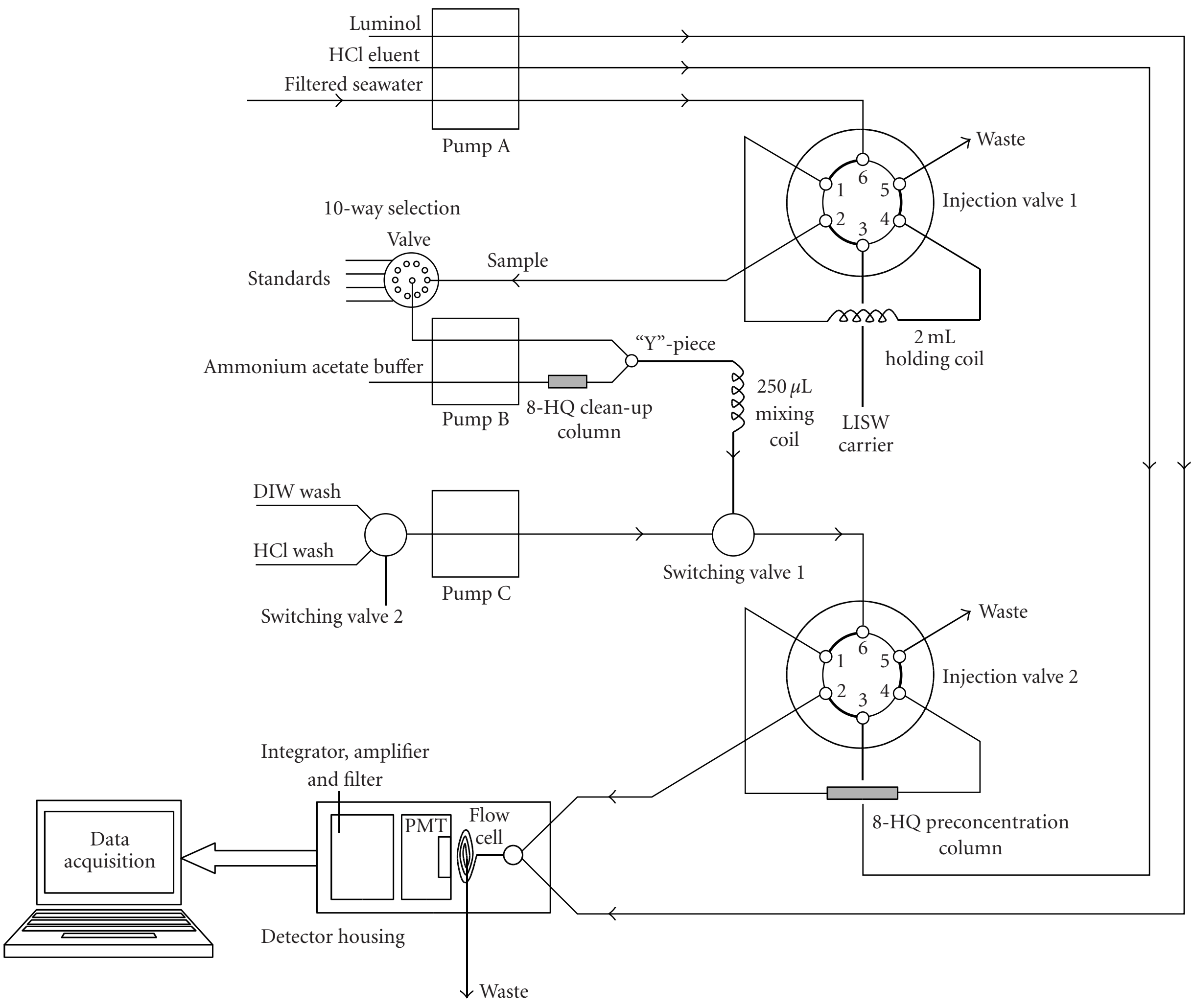

Figure 1: FI-CL manifold for the determination of iron in seawater.

stock pre-cleaned through an 8-HQ column. The eluent was 0.09 M Q-HCl and the strong acid wash (used only periodically to clean the manifold) was 0.6 M Q-HCl. Low iron seawater (LISW) obtained from the open ocean was used as the carrier stream to transport the sample from the holding loop to the preconcentration column.

### 2.2. FI manifold

Figure 1 shows the automated FI-CL manifold. Pumps A, B, and C were 4-channel peristaltic pumps (Gilson Minipuls 3, Anachem, Luton, UK). Injection valves 1 and 2 were ¼-28, 6-port, low-pressure valves (Cheminert C22, Valco, Houston, USA) with two position microelectronic actuation. A ¼-28, 10-port, low-pressure selection valve (Cheminert C25, Valco, Houston, USA) with multiposition micro-electronic actuation was used to switch between standards and the sample. Switching valves were PTFE 3-way, two-position solenoids (EW-01367-72, Cole-Parmer, Hanwell, UK). Pumps and valves were operated at 5 V dc (TTL) and switches at 12 V dc. A power saver relay reduced the solenoid input voltage to 8 V dc when energising for extended periods.

The detection system was a coiled transparent PVC flow cell (1.0 mm i.d.) mounted on the side window of a 5 V dc photon counting head (model H6240-01, Hamamatsu Photonics, Welwyn Garden City, UK). Detector specifications are given in Table 1. The TTL pulse train from the photon counting head was integrated, amplified, and filtered prior to data acquisition (Figure 2).

Flow lines, fittings, and connectors were cleaned for 1 day with 0.6 M Q-HCl and DIW prior to use. Manifold tubing was 0.75 mm i.d. PTFE (Fisher Scientific, Loughborough, UK). Peristaltic pump tubing was flow-rated PVC (Elkay, Basingstoke, UK). Preconcentration, matrix elimination, and sample buffer clean-up was performed in line using 8-hydroxyquinoline (8HQ) immobilised on a vinyl copolymer resin packed into 50 $\mu$L micro-columns [8].

Clean surface seawater was supplied to the FI manifold at sea using a high-volume peristaltic pump (7591-00, Cole Palmer Instrument Co.) connected to a torpedo-shaped fish,

TABLE 1: Specifications of miniature photon counting head.

| Maximum ratings | | |
|---|---|---|
| Parameter | Value | Unit |
| Supply voltage | +6 | V dc |
| Operating temperature range | +5 to 40 | °C |
| Storage temperature range | −20 to 50 | °C |
| Specifications at 25°C | | |
| Parameter | H6240-01 | Unit |
| Effective area | 4 × 20 | $mm^2$ |
| Spectral response | 185 to 850 | nm |
| Typical dark count | 80 | cps |
| Maximum dark count | 200 | cps |
| Counting linearity | 2.5 | Mcps |
| Pulse pair resolution | 35 | ns |
| Output pulse width | 30 | ns |
| Output logic | TTL, positive | |
| Input voltage | +5 | V dc |
| Input current at 2.5 Mcps output | Maximum 80 | mA |

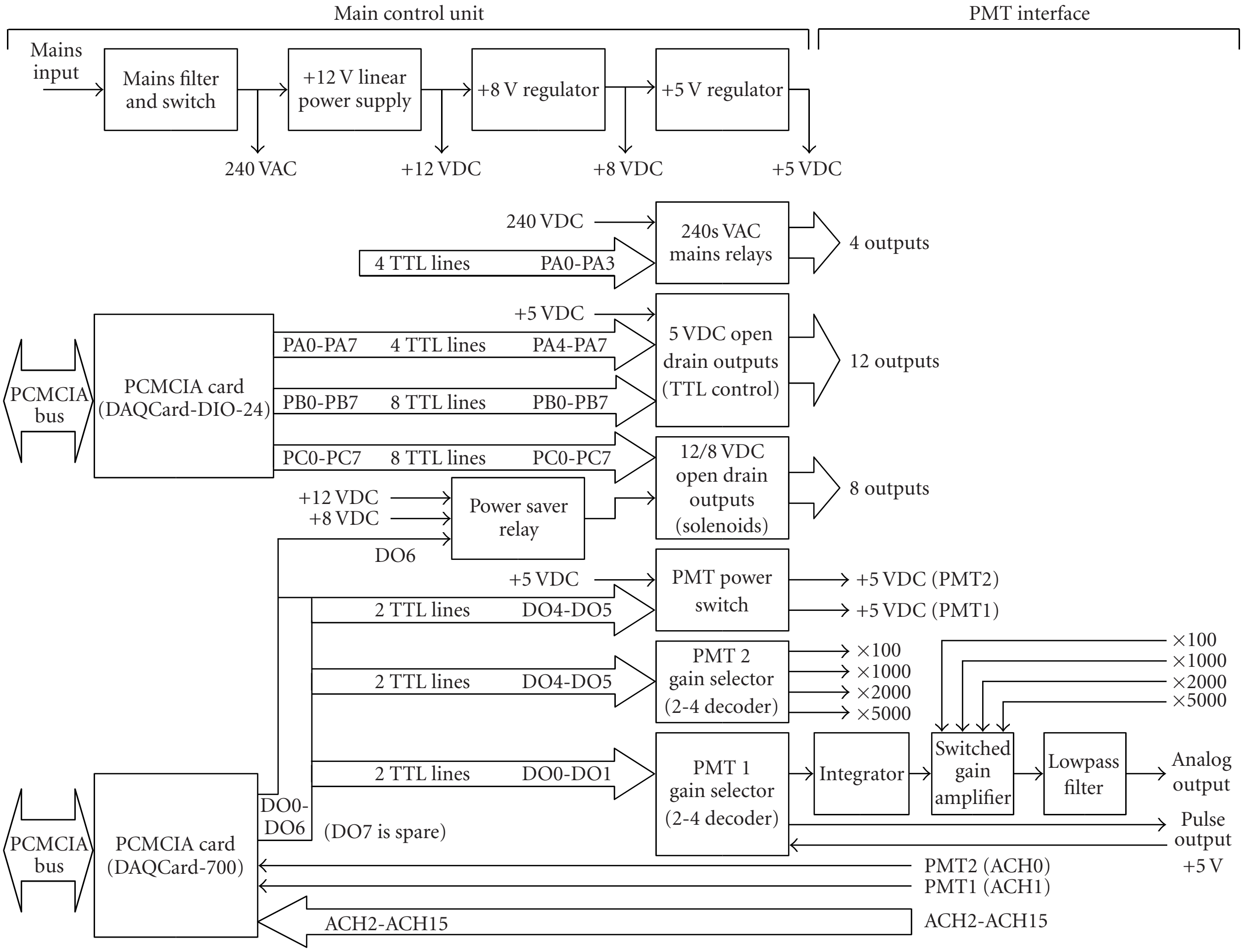

FIGURE 2: Block diagram of the automated FI-CL instrument incorporating the main control unit and PMT interface (integrator, amplifier, and filter are shown on PMT 1 only).

Table 2: Timing sequence for one analytical cycle. The pump and valve numbers refer to those shown in Figure 1.

| Elapsed time (s) | Pumps | | | Injection valves[a] | | Switching valves | | 10-way selection valve position[c] | Operation |
|---|---|---|---|---|---|---|---|---|---|
| | A | B | C | 1 | 2 | 1 | 2[b] | | |
| 0 | On | On | Off | On | Off | On | Off | 1 | Load |
| 60 | On | Off | On | Off | Off | Off | Off | 1 | Wash |
| 100 | On | Off | Off | Off | On | Off | Off | 1 | Elute |
| 160 | On | Off | On | Off | Off | Off | Off | 1 | Rinse |
| 180 | Cycle back to line 1 | | | | | | | | |

[a]Injection valves: Off=load sample and On=elute sample.

[b]Switching valve 2 is On only when an acid wash solution is passed over the 8HQ column.

[c]10-way selection valve remains in position 1 (sample port). For calibration this valve switches to positions 2–10 (depending on the number of standards to be run).

which was towed alongside the research vessel at a depth of 1–2 m below the surface, 5 m from the ship's hull. For water column samples, seawater was collected in acid-washed polycarbonate samplers suspended off Kevlar hydroline, following standard trace metal sampling methods [12]. Seawater was filtered in-line through a 0.4 $\mu$m cellulose acetate membrane contained in a polypropylene cartridge unit (Sartorius, Epsom, UK). Samples for iron(II) determinations were fed directly to the analyser at ambient seawater pH. Samples for iron(II+III) were acidified to pH $\sim$ 2 with Q-HCl and reduced off-line using 100 $\mu$M $Na_2SO_3$ (4 h) prior to analysis.

One complete analytical cycle, consisting of sample load, DIW rinse and elution, took 3 minutes. The operation, the state of each component (on or off for switching and injection valves; position for selection valve), and associated timing parameters during each cycle are shown in Table 2.

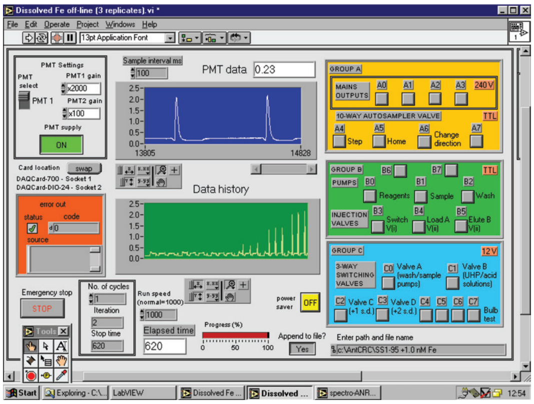

Figure 3: LabVIEW graphical user front panel for the automated virtual instrument. The *PMT data* and *Data history* displays show the unit in calibration mode.

### 2.3. Interface

Instrument control was achieved via a DAQCard-DIO-24 card (National Instruments Corp., Newbury, UK) with 24 digital input/output TTL lines, and signal acquisition was via a DAQCard-700, with 16-channel, 12-bit A/D conversion. This card was also used for changing PMT gain. Virtual instrument (VI) software (Ruthern Instruments Ltd., Bodmin, UK) was written in LabVIEW version 5.1 (National Instruments Corp.). The interface had two units, one for controlling pumps and valves and one for the PMT and signal processing (Figure 2). The LabVIEW VI front panel contained ready-to-use switches, buttons, controls, and graphical displays of detector readings (Figure 3). Each element in the front panel was connected via the wiring diagram (Figure 4), which included functions for signal processing, timing of operations, and file management.

### 3. RESULTS AND DISCUSSION

### 3.1. Detector performance

The PMT interface contained a 4-position switched gain amplifier (Figure 2). This provided settings of $\times 100$, $\times 1000$, $\times 2000$, and $\times 5000$, selectable by the control VI software, which allowed the sensitivity to be adjusted to suit the variable concentrations of iron found in seawater. The effect of each of these settings on the CL background emission, background noise, and analyte signal for a 2.0 nM iron(II) standard was investigated in direct injection mode (i.e., no preconcentration column), and the results are shown in Figure 5. The CL background noise (peak-to-peak) showed no change with gain setting, but both the CL emission for iron and the background CL emission increased linearly with respect to PMT gain. The maximum signal-to-noise ratio was obtained at the highest gain setting ($\times 5000$), which was therefore most suitable for iron-depleted open-ocean measurements. For environments with higher iron concentrations (such as coastal and estuarine waters), a lower gain setting can be used to provide an expanded linear range.

### 3.2. Analytical figures of merit

Figure 6 shows a typical FI trace for the blank, sample, and standard additions of 0.2–1.0 nM iron(II) spikes to a seawater sample. The mean repeatability and standard deviation for 4 replicates over this range was 5.9 ± 3.2%. The standard addition plot showed excellent linearity ($R^2 = 0.9979$) over this range. The iron(II) blank was typically $24 \pm 7$ pM ($n = 4$),

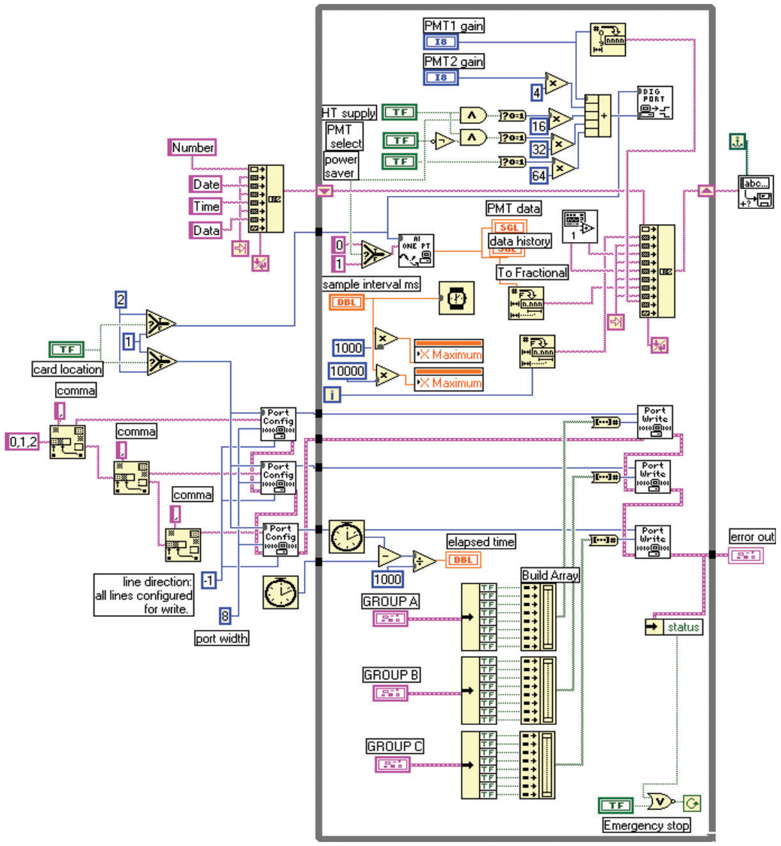

Figure 4: Wiring diagram showing the graphical code for instrument control and data acquisition. This code drives the functions shown on the front panel in Figure 3.

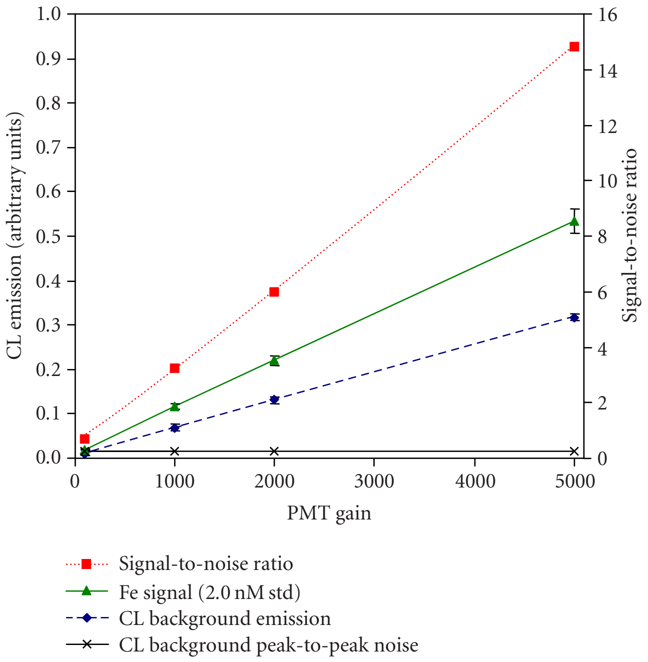

Figure 5: Effect of changing the PMT gain on the CL background emission, CL background peak-to-peak noise, analyte signal and signal-to-noise ratio with the instrument in direct injection mode (no preconcentration). Error bars indicate ±1 sd.

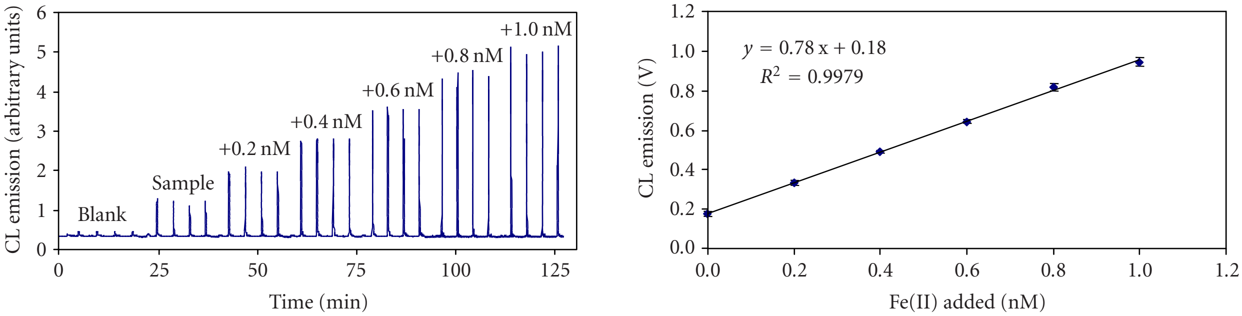

Figure 6: Shipboard calibration peaks and corresponding standard additions plot for iron over the range 0.2–1.0 nM. Error bars indicate ±1 sd.

resulting in a limit of detection of 21 pM (defined as three times the standard deviation of the blank). The major contributions to the blank signal were from iron impurities in the ammonium acetate buffer, DIW used for column washing, and (for iron(II+III) determinations) the acid and sulfite used for sample pretreatment [8].

### 3.3. Field validation

The optimised FI-CL instrument was trialled at a hydrocast station close to the Antarctic continent during a Southern Ocean expedition (November 2001) along the CLIVAR SR3 line (~ 141°E). Figure 7 shows the profiles of dissolved iron and temperature in the upper water column (25–300 m), illustrating the depletion of iron in the mixed layer (down to 100 m) due to biological uptake and its gradual regeneration at depth due to microbial decomposition of biogenic particles. Iron concentrations were between 220 and 360 pM at this location, consistent with literature data [13]. At sea, the instrument was totally reliable over 50 days of near continuous use for surface transects and depth profiling, with no downtime in spite of the harsh conditions experienced in this environment. A report on the environmental significance of the complete dataset from the 2001 CLIVAR SR3 expedition, obtained using this instrumentation, will be presented elsewhere.

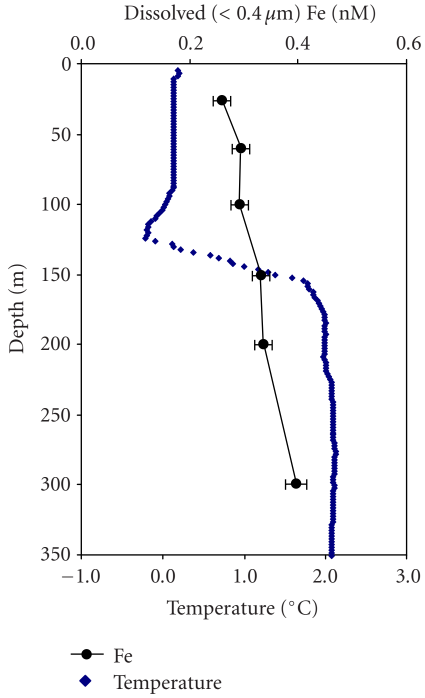

Figure 7: Typical depth profiles of dissolved iron and temperature in the upper water column of the Southern Ocean south of Australia at 141° E, 61° S. Error bars indicate ±1 sd.

### 4. CONCLUSIONS

The automated virtual instrument uses flow injection with chemiluminescence detection for the determination of iron in seawater. This is an inexpensive, portable, and robust system suitable for shipboard deployment. The detection limit of 21 pM allows the determination of iron in all marine environments, including remote, iron-limited open-ocean regions. In addition, the use of off-the-shelf components and industry standard graphical programming software makes the instrument readily adaptable to related analytes (e.g., cobalt [14], copper [15]) using well documented chemiluminescence reactions. This instrumentation should be easily transferable between laboratories, thus facilitating the harmonisation of analytical methods for the determination of iron in seawater, a current initiative of the Scientific Committee for Oceanic Research (SCOR) Working Group 109 (Biogeochemistry of Iron in Seawater) (http://www.jhu.edu/~scor/wg109front.htm).

### ACKNOWLEDGMENTS

The authors thank John Wood (Ruthern Instruments) for constructing the main control unit and PMT interface. This work was funded by the EU programme MEMOSEA (Trace Metal Monitoring in Surface Marine Waters and Estuaries, MAS3-CT97-0143, NERC Grant NER/A/S/2003/00489) and an Australian Research Council IREX Fellowship to A. R. Bowie (X00106765).

### REFERENCES

[1] R. J. Geider and J. La Roche, "The role of iron in phytoplankton photosynthesis and the potential for iron-limitation of primary productivity in the sea," *Photosynthesis Research*, vol. 39, pp. 275–301, 1994.

[2] P. W. Boyd, A. J. Watson, C. S. Law, et al., "A mesoscale phytoplankton bloom in the polar southern ocean stimulated by iron fertilization," *Nature*, vol. 407, no. 6805, pp. 695–702, 2000.

[3] P. Falkowski, R. J. Scholes, E. Boyle, et al., "The global carbon cycle: a test of our knowledge of earth as a system," *Science*, vol. 290, no. 5490, pp. 291–296, 2000.

[4] K. S. Johnson, R. M. Gordon, and K. H. Coale, "What controls dissolved iron concentrations in the world ocean?" *Marine Chemistry*, vol. 57, no. 3-4, pp. 137–161, 1997.

[5] J. F. Wu and E. A. Boyle, "Determination of iron in seawater by high-resolution isotope dilution inductively coupled plasma mass spectrometry after $Mg(OH)_2$ coprecipitation," *Analytica Chimica Acta*, vol. 367, no. 1–3, pp. 183–191, 1998.

[6] M. L. Wells and K. W. Bruland, "An improved method for rapid preconcentration and determination of bioactive trace metals in seawater using solid phase extraction and high resolution inductively coupled plasma mass spectrometry," *Marine Chemistry*, vol. 63, no. 1-2, pp. 145–153, 1998.

[7] K. W. Bruland, R. P. Franks, G. A. Knauer, and J. H. Martin, "Sampling and analytical methods for the determination of copper, cadmium, zinc, and nickel at the nanogram per liter level in seawater," *Analytica Chimica Acta*, vol. 105, pp. 233–245, 1979.

[8] A. R. Bowie, E. P. Achterberg, R. F. C. Mantoura, and P. J. Worsfold, "Determination of sub-nanomolar levels of iron in seawater using flow injection with chemiluminescence detection," *Analytica Chimica Acta*, vol. 361, no. 3, pp. 189–200, 1998.

[9] J. T. M. de Jong, J. den Das, U. Bathmann, et al., "Dissolved iron at subnanomolar levels in the southern ocean as determined by ship-board analysis," *Analytica Chimica Acta*, vol. 377, no. 2-3, pp. 113–124, 1998.

[10] C. I. Measures, J. Yuan, and J. A. Resing, "Determination of iron in seawater by flow injection analysis using in-line preconcentration and spectrophotometric detection," *Marine Chemistry*, vol. 50, no. 1–4, pp. 3–12, 1995.

[11] H. Obata and C. M. G. van den Berg, "Determination of picomolar levels of iron in seawater using catalytic cathodic stripping voltammetry," *Analytical Chemistry*, vol. 73, no. 11, pp. 2522–2528, 2001.

[12] P. N. Sedwick, P. R. Edwards, D. J. Mackey, F. B. Griffiths, and J. S. Parslow, "Iron and manganese in surface waters of the Australian subantarctic region," *Deep-Sea Research I*, vol. 44, no. 7, pp. 1239–1253, 1997.

[13] H. J. W. de Baar and J. T. M de Jong, "Distributions, sources and sinks of iron in seawater," in *The Biogeochemistry of Iron in Seawater*, D. R. Turner and K. A. Hunter, Eds., chapter 5, pp. 123–254, John Wiley & Sons, Chichester, UK, 2001.

[14] V. Cannizzaro, A. R. Bowie, A. Sax, E. P. Achterberg, and P. J. Worsfold, "Determination of cobalt and iron in estuarine and coastal waters using flow injection with chemiluminescence detection," *Analyst*, vol. 125, pp. 51–57, 2000.

[15] E. P. Achterberg, C. B. Braungardt, R. C. Sandford, and P. J. Worsfold, "UV digestion of seawater samples prior to the determination of copper using flow injection with chemiluminescence detection," *Analytica Chimica Acta*, vol. 440, no. 1, pp. 27–36, 2001.

